# Microarray analysis of defined *Mycobacterium tuberculosis *populations using RNA amplification strategies

**DOI:** 10.1186/1471-2164-9-94

**Published:** 2008-02-25

**Authors:** Simon J Waddell, Ken Laing, Claire Senner, Philip D Butcher

**Affiliations:** 1 Medical Microbiology, Centre for Infection, Division of Cellular & Molecular Medicine, St. George's University of London, Cranmer Terrace, Tooting, London, UK

## Abstract

**Background:**

The amplification of bacterial RNA is required if complex host-pathogen interactions are to be studied where the recovery of bacterial RNA is limited. Here, using a whole genome *Mycobacterium tuberculosis *microarray to measure cross-genome representation of amplified mRNA populations, we have investigated two approaches to RNA amplification using different priming strategies. The first using oligo-dT primers after polyadenylation of the bacterial RNA, the second using a set of mycobacterial amplification-directed primers both linked to T7 polymerase *in vitro *run off transcription.

**Results:**

The reproducibility, sensitivity, and the representational bias introduced by these amplification systems were examined by contrasting expression profiles of the amplified products from inputs of 500, 50 and 5 ng total *M. tuberculosis *RNA with unamplified RNA from the same source. In addition, as a direct measure of the effectiveness of bacterial amplification for identifying biologically relevant changes in gene expression, a model *M. tuberculosis *system of microaerophilic growth and non-replicating persistence was used to assess the capability of amplified RNA microarray comparisons. Mycobacterial RNA was reproducibly amplified using both methods from as little as 5 ng total RNA (~equivalent to 2 × 10^5 ^bacilli). Differential gene expression patterns observed with unamplified RNA in the switch from aerobic to microaerophilic growth were also reflected in the amplified expression profiles using both methods.

**Conclusion:**

Here we describe two reproducible methods of bacterial RNA amplification that will allow previously intractable host-pathogen interactions during bacterial infection to be explored at the whole genome level by RNA profiling.

## Background

The application of bacterial transcriptomics to complex biological situations is currently hampered by the low amounts of bacterial RNA available after purification from *in vitro *and *in vivo *models of infection. In contrast to eukaryotic mRNA, techniques to amplify bacterial RNA for use in microarray analyses have not been available until recently, and crucially have not been evaluated for *M. tuberculosis *in well characterised models in which differential gene expression is well described and validated. Many of the established eukaryotic amplification strategies have been based on the Eberwine method of *in vitro *transcription with T7 RNA polymerase using oligo-dT primers to selectively amplify eukaryotic mRNA containing poly-A tails [1]. However due to the limited poly-adenylation of bacterial transcripts [2], this technique is not suitable for amplifying bacterial RNA [3]. Lately a number of methods based on the Eberwine T7-transcription system have been adapted for bacterial RNA amplification using either oligo-dT [4-7] or random [8,9] priming approaches.

In this study we examine two different methods of bacterial RNA amplification using alternative priming strategies, on the basis that priming efficiencies will have maximum effect on the representation of mRNA populations through possible priming bias. Whole genome representation of the amplified products was measured using a *Mycobacterium tuberculosis *microarray. The first priming method involved polyadenylating the bacterial RNA prior to amplification using the Eberwine T7-oligo-dT method. The second priming strategy used a set of mycobacterial amplification-directed primers (with the addition of a T7 promoter sequence) to prime the first strand cDNA synthesis instead of oligo-dT (as in the eukaryotic system) followed by T7 transcript amplification. A minimal set of amplification-directed primers (ADP) was designed to prime all the genes in the combined genomes of *M. tuberculosis *H37Rv [10], CDC1551 [11], and *M. bovis *AF2122/97 [12] using a process first described by Talaat *et al*. [13], and which have recently been used in conjunction with a template switching strategy for bacterial RNA amplification [14-16].

A number of variables were investigated: size distribution and yield of amplified product, the sensitivity and reproducibility of the amplification systems, the impact of contaminating genomic DNA and eukaryotic RNA, and the mRNA representation bias introduced by amplification. To explore these parameters three experimental designs were required: (A) Amplified and unamplified RNA from the same source (*M. tuberculosis *grown aerobically *in vitro*) were compared to measure the reproducibility and bias introduced by RNA amplification; (B) RNA extracted from *M. tuberculosis *cultures under well characterised aerobic and microaerophilic (NRP1) conditions [17,18] were contrasted using amplified and unamplified RNA to assess the biological veracity of amplified RNA transcriptional patterns. We argue that the use of amplification methods to define transcriptomes of bacteria in environments such as infected tissue where the pattern of differential gene expression may well be significantly altered can only be reliably interpreted with an evaluation of any bias introduced by the amplification process. This cannot be assessed in an undefined environment likely to induce significant differential transcription. Hence we have chosen a well-established model in which transcriptome patterns have been established by ourselves and other groups [19,18]. (C) Finally the impact of contaminating eukaryotic RNA on amplified mycobacterial gene expression patterns was determined by comparing profiles derived from mixed RNA populations. The use of both direct comparisons and a model system allowed the reliability and representative nature of amplified RNA as well as the meaningful biological interpretation of amplified transcriptomes to be explored.

## Results

### Experimental approach

The application of bacterial RNA amplification methods to explore previously inaccessible models of infection by microarray analysis was assessed using three experimental strategies. In the first (experimental design A), unamplified and amplified mycobacterial RNA (extracted from agitated mid-log phase bacilli) were hybridised to a *M. tuberculosis *microarray against a genomic DNA reference. This strategy allowed the variation and bias introduced by amplification into the gene expression profiles of the same starting RNA to be examined. A second microarray study (experimental design B) was used to identify significantly differentially expressed genes by hybridising amplified RNA from aerobic and microaerophilic (NRP1) culture conditions directly against one another. These significantly differentially expressed genes were then compared to patterns previously determined by microarray analysis from unamplified RNA and from the published literature as a measure of how well biologically relevant transcriptional profiles were represented in the amplified datasets. A third strategy comparing the mycobacterial gene expression profiles derived from mixed RNA populations, where bacterial RNA may be contaminated with eukaryotic host RNA, was used to determine the effect of co-purified eukaryotic RNA on the amplified mycobacterial transcriptional profile. This scenario may be relevant to *in vivo *models of infection such as experimental murine tissue or human-derived tissue in which bacterial RNA represents a minor component of total RNA.

The addition of non-template sequence to a primer may adversely modify the stability of a primer-template dimer; we wished therefore to assess the effect of adding a T7 promoter and additional degenerate linker sequence to a specific primer. We measured the specificity and efficacy of the mycobacterial amplification-directed primers by microarray analysis comparing the RNA profile generated after priming with individual 7 nt ADP-specific primer sequences with that primed using the respective 40 nt ADP with additional T7 promoter and degenerate sequence. Using cutoffs of 2 fold signal/background or a signal > 500 to identify successful priming of genes during the RT-labelling reactions, an average of 92–93% of genes (identified from the analysis of 5 ADPs +/- T7 sequences) primed with the ADP+T7 sequence were also primed by the ADP-specific 7 nt primer alone. In addition, the average Spearman's rank correlation of genes identified by either cutoff (2 fold signal/background or signal > 500) to be primed by (x5) ADP +/- T7 sequence was 0.89–0.91. The priming specificity of the ADP primers therefore was not significantly altered by the addition of the T7 sequences, consequently the ADP primers were considered suitable to use as *M. tuberculosis *amplification-directed primers.

### Unamplified vs. amplified RNA comparison

500, 50 and 5 ng of total RNA extracted from aerated mid-log phase *M. tuberculosis *was amplified using the ADP or oligo-dT priming methods and hybridised to a *M. tuberculosis *microarray. The performance of these amplification systems was measured by contrasting the yield, minimum input RNA, reproducibility, and how representative the amplified expression profile was compared to unamplified RNA.

#### Amplified RNA size distribution and yield

**Figure 1:**
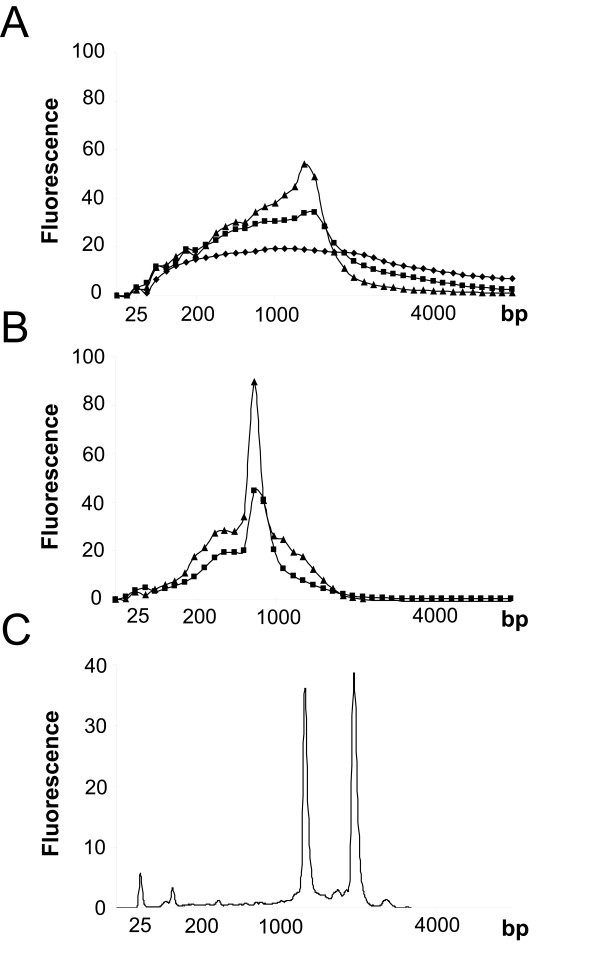
The size distribution of products amplified from 500 (triangle), 50 (square), 5 ng (diamond) *M. tuberculosis *total RNA using oligo-dT (A) and ADP (B) amplification strategies. (C) Unamplified total mycobacterial RNA. The peaks at 25 bp represent the marker added to all samples. The size distributions were plotted using the average of 2–4 replicate amplifications. Abundance units are detailed in relative fluorescence and plotted against migration time that has been converted into a base pair (bp) estimate of product size as measured using the Agilent Bioanalyser.

From total RNA inputs of 500, 50 and 5 ng, average yields of 91–198 μg aRNA were generated from a single round of amplification using the oligo-dT and ADP methods (Table 1), representing an amplification of between 10^2^-10^4 ^fold total RNA depending on priming strategy and input amount. No product however was detected from 5 ng input total RNA using the ADP priming strategy. Both amplification methods therefore (except for ADP at 5 ng) generated sufficient amplified product for microarray analysis. The size distribution of the aRNA populations differed in modal product size which varied with amplification method and input RNA amount (Figure 1A and 1B), with approximate modal product sizes of 1600 and 750 bp from 500 ng input RNA using oligo-dT and ADP methods respectively. The size distribution of amplified RNA therefore differs markedly from unamplified total RNA that is dominated by 16s and 23s ribosomal peaks (Figure 1C). Amplified product was generated from 500 ng genomic DNA using both methods, indicating that both strategies were capable of priming in the presence of DNA. Genomic DNA however was removed by DNase treatment during RNA extraction to minimise this effect. To confirm that product generated from DNase-treated total RNA preparations were from RNA, 500 ng of DNase-treated and then RNase-treated total RNA showed little or no amplified product on the Bioanalyser, suggesting minimal influence on RNA profiles from contaminating genomic DNA in the RNA preparations used in this study. Additionally there was no detectable product generated from 500 ng mycobacterial total RNA using the oligo-dT T7-based amplification method without first polyadenylating the nucleic acid, indicating the absence of polyA or poor oligo-dT priming from existing polyA in these samples.

**Table 1:** Mean aRNA yields (μg) using the oligo-dT and ADP amplification methods from total RNA inputs of 500, 50 and 5 ng. Average yield was determined by spectrophotometric analysis from 2–4 replicate amplification reactions. The range of amplified RNA yields is also detailed.

Amp Method Yield (μg)	Amount of input RNA (ng)		
	
	5	50	500
Oligo-dT	130 (+/- 12)	178 (+/- 2)	198 (+/- 35)
ADP	-	91 (+/- 2)	149 (+/- 18)

#### Reproducibility

The reproducibility of these methods was tested by amplifying 500 ng RNA in duplicate on two occasions to measure the variation in yield, size distribution and gene expression profile of products from different amplification reactions. The yield (Table 1) and the size distribution of products from each method were highly reproducible between amplifications. In addition, the correlation coefficients of the ranked signal of all genes from unamplified RNA and RNA amplified using the oligo-dT and ADP methods were calculated to be 0.91, 0.97 and 0.86 respectively between replicate samples amplified and hybridised on the same day. For samples amplified and hybridised on different days the correlation coefficients for the unamplified RNA, oligo-dT and ADP methods respectively were 0.87, 0.96, 0.85. Thus the amplification of RNA on different occasions added little to the variation inherent in microarray analyses.

#### Representation

The differential amplification of RNA sequences may re-order the ranked intensities of genes on the array and result in a biased description of the RNA population. The effect of this would be over- or under-representation of some amplified message in comparison with unamplified. Biased representation could also occur by a failure to sufficiently amplify specific mRNA transcripts that exist below a detection threshold, such as very low abundance mRNA or mRNA with low hybridisation signals on the array due to a number of factors independent of amplification. In either instance, a potential loss or misrepresentation of information could result from a comparison of amplified vs. unamplified RNA.

**Figure 2:**
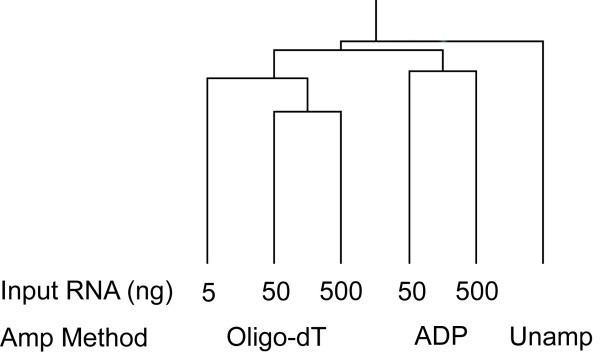
Spearman's rank correlation tree of the gene expression patterns from amplified and unamplified RNA. RNA was amplified from 500, 50 and 5 ng total RNA using the oligo-dT and ADP methods. All *M. tuberculosis *H37Rv genes were clustered using median expression ratios from 2–6 replicate hybridisations.

Bias effects were examined in three ways; firstly, using Spearman's rank correlation to compare the signal intensities of all genes for both methods (and input amount) against all others (including unamplified); secondly, by using an arbitrary 2.5 fold cut-off to identify gross differences in the signal ratio of amplified to unamplified; and thirdly, by comparing the number of genes below a signal threshold to identify the potential loss of information from RNA amplification. A correlation matrix comparing the ranked mean signal of each gene from the amplified products for each amplification strategy with input of 500, 50 and 5 ng of RNA with unamplified RNA was performed (Table 2). The amplification-directed primer approach using 500 ng input (0.840) and oligo-dT method at 500 (0.839), 50 (0.838) and 5 ng (0.838) were all similarly correlated to the unamplified RNA profile. The correlation coefficient of the ADP amplification method from 50 ng input RNA to unamplified RNA was lower (0.788). This pattern is reflected in the clustering of the expression ratios of all genes to the DNA universal reference (Figure 2). The correlation between RNA amplified by the same method but using different starting amounts was high, a mean of 0.965 using the oligo-dT method and 0.895 using the ADP amplification method (Table 2); illustrating consistent amplification with both strategies irrespective of the starting amount of RNA.

**Table 2:** A matrix describing the correlation between mean gene expression profiles of amplified and unamplified *M. tuberculosis *total RNA from 500, 50 and 5 ng starting amounts. Spearman's rank correlations were calculated from the mean RNA signals from all *M. tuberculosis *H37Rv genes. Correlations in bold type highlight the relationship between differing input amounts of RNA using the same amplification method, oligo-dT or ADP. The correlations between unamplified (Unamp) and amplified products are marked in italics.

Sample		Oligo-dT	ADP	Oligo-dT	ADP	Oligo-dT
	
	(ng input)	5	50	50	500	500
ADP	50	0.865				
Oligo-dT	50	**0.964**	0.894			
ADP	500	0.861	**0.895**	0.891		
Oligo-dT	500	**0.949**	0.891	**0.982**	0.902	
Unamp	-	*0.838*	*0.788*	*0.838*	*0.840*	*0.839*

A second measure of the similarity between amplified and unamplified RNA was to determine the number of genes over or under-represented in amplified compared to unamplified expression profiles. Using a 2.5 fold cutoff the ADP method had the smallest number of genes over or under represented (635 at 500 ng input RNA, 979 at 50 ng) compared to unamplified RNA. Using the oligo-dT strategy 907, 951 and 800 genes were identified to be 2.5 fold over or under represented after amplification with starting amounts of 500, 50 and 5 ng respectively.

**Figure 3:**
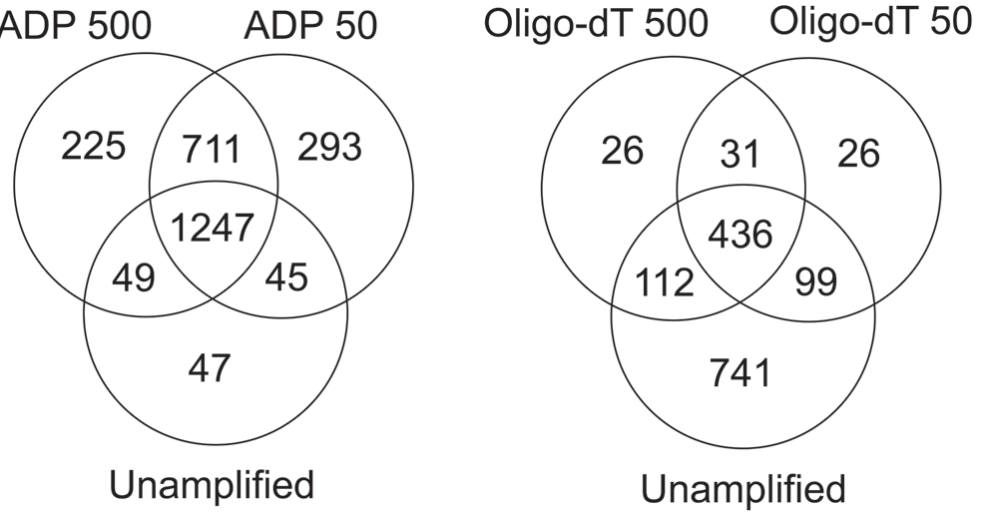
A comparison of the number of genes not detected by microarray analysis from unamplified RNA or from products generated by oligo-dT and ADP amplification methods using 500 or 50 ng starting total RNA. The number of genes below a 2 fold signal/background threshold in the RNA channel was compared between amplified and unamplified gene expression signatures as a measure of the potential transcriptome data lost during amplification.

A third parameter was considered to assess the potential problem that genes may be poorly amplified by the respective amplification system to an extent that the signals were not detected by microarray analysis resulting in the loss of data. The number of genes under a 2 fold median signal/background ratio in the RNA channel was compared between amplified and unamplified samples. The smallest number of 'absent' genes below this detection threshold was 605 and 592 (500 and 50 ng of input RNA respectively) using the oligo-dT amplification method. However the number of undetected genes nearly trebled with a further reduction of input RNA to 5 ng using the same amplification strategy, increasing the number of genes below the background threshold to 1606. A greater number of genes, 2232 and 2296, were not detected using the ADP method at 500 and 50 ng of input respectively. A comparison of genes not detected by microarray analysis from 500 and 50 ng input RNA for each method together with the unamplified RNA controls is depicted in Figure 3. In all cases the majority of genes that were not detected by microarray analysis using amplified RNA overlapped with those determined to be under the detection threshold (1388 genes) using unamplified RNA.

Thus, both amplification systems were applicable to microarray analysis in both yield and reproducibility; both methods were also representative of the unamplified RNA population to a large extent (with correlation coefficients of approximately 0.8) although differences in RNA profile could be detected that may detrimentally affect direct comparisons of amplified to unamplified RNA. Although useful for investigating the accuracy of these bacterial amplification systems, the above experimental design examining unamplified and amplified RNA from the same source did not allow for a comparison of amplification methods in a microarray experiment designed to extract meaningful biological information. Therefore *M. tuberculosis *RNA extracted under well-defined aerobic and microaerophilic growth conditions was amplified and compared. Significant differences in gene expression identified from this model system were evaluated against a comparison of unamplified RNA and published data.

### Aerobic vs. NRP1 comparison

The changes in transcriptome profile of *M. tuberculosis *H37Rv were determined in aerobic growth compared to growth under conditions of limited oxygen (non-replicating persistence, NRP1). These microaerophilic conditions were chosen because the experimental procedures have been previously defined [17] and a subset of genes has consistently been identified to be induced during NRP [19,18]. In order to measure the ability of amplified RNA microarray comparisons to successfully interpret biologically relevant changes in gene expression; 500, 50 or 5 ng of aerobic and NRP1 mycobacterial total RNA was amplified using either the amplification-directed primer (ADP) or oligo-dT amplification methods and co-hybridised in a direct comparison of amplified vs. amplified RNA for the respective priming methods. Genes identified to be significantly differentially expressed (with a t-test p-value < 0.05 with Benjamini and Hochberg multiple testing correction, and a fold change > 2.5) were compared with gene lists derived from unamplified RNA and with the published literature [19,18].

#### Comparison of genes induced in microaerophilic conditions

**Figure 4:**
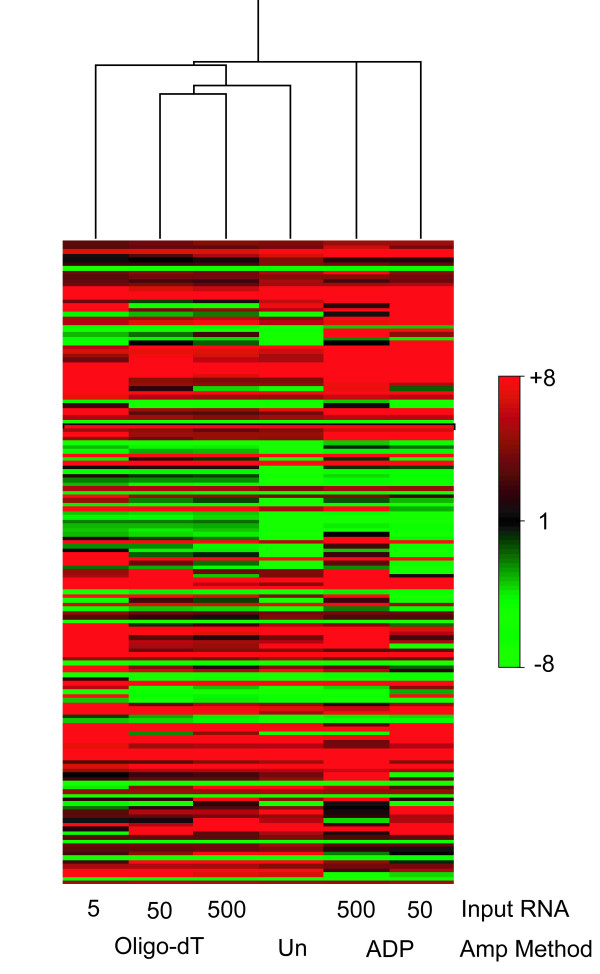
A Spearman's rank correlation of the 155 genes identified to be significantly differentially expressed in microaerophilic compared to aerobic *M. tuberculosis *growth conditions using unamplified RNA (marked Un). The mean gene expression ratios derived from unamplified RNA and from the products of 500, 50, and 5 ng amplifications using oligo-dT and ADP methods are displayed. Genes are ordered in rows, amplification conditions as columns. Red colouring indicates genes induced in microaerophilic vs. aerobic growth conditions; green colouring denotes repression.

91, 61 and 20 genes were identified as significantly induced in NRP1 compared to aerobic growth using the oligo-dT amplification method using 500, 50 and 5 ng of RNA in the amplifications. Whist 24 and 42 genes were significantly induced in the comparison using ADP amplification from 500 ng and 50 ng input RNA respectively. These gene lists were compared with a gene list of 100 significantly induced genes derived via the same statistical measures in an aerobic vs. NRP1 comparison using unamplified RNA. Of the 100 genes identified as induced in the unamplified comparison 61 overlapped with the significant genes derived from the 500 ng oligo-dT analysis, 40 from 50 ng and 13 from 5 ng oligo-dT amplification. 11 genes overlapped with the significantly induced gene list from the ADP 500 ng microarray analysis, and 21 from ADP amplification of 50 ng input RNA. The oligo-dT amplification strategy therefore correlated most closely with the changes in gene expression identified using unamplified RNA extracted from *M. tuberculosis *in microaerophilic compared to aerobic growth conditions. Furthermore the number of significantly induced genes declined as the input RNA decreased from 500 to 50 to 5 ng using this amplification method. This pattern is further illustrated in Figure 4 by clustering the 155 genes identified to be significantly differentially expressed in the unamplified aerobic vs. NRP1 microarray comparison with the amplified expression dataset. The majority of genes significantly induced in the unamplified comparison that were missed in the amplified comparisons were excluded on t-test p-value; this is likely due to the effect of lower signal intensities increasing the variation of gene expression ratios and thus decreasing the t-test significances in the amplified RNA comparisons.

#### Biological significance

The biological patterns of gene expression associated with the shift from aerobic to microaerophilic growth could be readily identified using amplified RNA. The hypergeometric function, that statistically tests for the probability that functional categories of genes are significantly enriched, was used to determine whether previously defined changes in gene expression pattern were identifiable in the gene lists generated in the aerobic vs. NRP1 comparisons using amplified RNA. Genes previously observed to be induced during non-proliferating conditions [18] were enriched in this comparison of aerobic vs. microaerophilic (NRP1) by probabilities of 6.5 × 10^-41 ^using unamplified RNA, 3.5 × 10^-32^, 9.6 × 10^-24^, and 8.2 × 10^-7 ^using the oligo-dT amplification method (500, 50, 5 ng input respectively), and 0.15, 1.9 × 10^-7 ^using the ADP amplification strategy. Similarly the *devR *(*dosR*) regulon previously identified to be induced by hypoxia [19] was also significantly represented in the genes induced by microaerophilic growth conditions with hypergeometric p-values of 1.6 × 10^-71 ^using unamplified RNA, 5.2 × 10^-47^, 1.0 × 10^-28 ^and 3.4 × 10^-6 ^in the oligo-dT comparison (500, 50 and 5 ng input respectively), and 0.26, 1.2 × 10^-9 ^in the ADP amplified comparisons. The hypergeometric probabilities for the ADP comparisons were significant only at an input of 50 ng total RNA; this may reflect suboptimal performance of the reverse transcription and a need to optimise the primer:template ratio. However, biologically significant patterns of gene expression may be identified by microarray analysis of amplified RNA from as low as 5 ng total RNA input.

### Impact of contaminating eukaryotic RNA

The studies above used purified total *M. tuberculosis *RNA as input into the two amplification systems, analogous to RNA extracted from *in vitro *conditions or from infection models where a differential lysis method of extraction (in which purified total bacterial RNA may be separated from eukaryotic RNA during extraction [20-22]) is applicable. However the extraction of eukaryotic alongside bacterial RNA is unavoidable with many bacteria or from some infection models. To determine any possible adverse effect of contaminating eukaryotic RNA on mycobacterial RNA amplification 250 ng mycobacterial RNA was amplified (in triplicate) using ADP or oligo-dT methods in the presence or absence of 250 ng eukaryotic RNA. The gene expression profiles in the presence or absence of eukaryotic RNA spikes were compared using a t-test (p-value < 0.05 with Benjamini and Hochberg multiple testing correction). Only 4 genes were identified to have differential transcript abundance after amplification using the oligo-dT method (all < 1.5 fold change), and no genes had significantly different RNA abundances after ADP amplification in the presence of contaminating eukaryotic RNA. In addition, the average Spearman's rank correlations comparing the mycobacterial transcriptional profiles derived from amplifications with and without eukaryotic RNA was 0.97 for oligo-dT amplified RNA, and 0.92 for RNA amplified using the ADP strategy. In an alternative analysis approach, 250 ng eukaryotic RNA alone was amplified using the oligo-dT and ADP methods and hybridised to the *M. tuberculosis *microarray. The detection of genes identified from these eukaryotic RNA hybridisations to have signal ratios > 2 fold over background did not change between *M. tuberculosis *RNA amplified alone or with contaminating eukaryotic RNA using either amplification system, (average gene expression ratio of 1.02 between *M. tuberculosis *amplifications +/- eukaryotic RNA). The impact therefore of contaminating levels of eukaryotic RNA after amplification (using either method) and hybridisation to a *M. tuberculosis *microarray was minimal. This correlates with the findings of Lawson and Johnston [15] using Linear Amplification of Prokaryotic Transcripts (LAPT) for bacterial amplification to investigate mixed RNA populations.

## Discussion

*M. tuberculosis *total RNA was successfully amplified and analysed by microarray analysis from between 5 to 500 ng of total RNA. The oligo-dT method, based on the polyadenylation of RNA followed by oligo-dT primed amplification, consistently gave the greatest yield of aRNA. The ADP method, using a subset of primers designed to initiate cDNA synthesis within the terminal region of all coding sequences within the *M. tuberculosis *H37Rv, CDC1551 and *M. bovis *AF2122/97 genomes, generated similar yields with 500 or 50 ng input RNA but failed to provide any significant product from 5 ng of input RNA. This may reflect suboptimal performance of the reverse transcription and the need to optimise primer:RNA ratio since lowering the primer input for a fixed amount of RNA affected the size distribution of the amplified product using this amplification system (data not shown). This may also account for the greater biological significance of comparisons from an RNA input of 50 compared to 500 ng using the ADP method. The observed differences in modal size between the ADP (~750 bp) and oligo-dT (~1600 bp) methods can be explained by the initial priming event during cDNA synthesis since all other aspects of these techniques were the same. ADP primers were designed to initiate cDNA synthesis within 500 nucleotides of the 3' of the coding sequences of each gene, however primers could bind elsewhere in the ORF. Thus ADP priming will initiate cDNA synthesis at multiple points within the message, in contrast oligo-dT priming will only occur within the terminal polyadenylated tails added to the 3' end of the polycistronic bacterial message (or indeed intragenic polyA sequences); this pattern was also observed by Rachman *et al*. [14]. Therefore ADP primed amplification would be expected to yield a shorter modal length product than oligo-dT primed RNA amplification and this was indeed the case. The implication for first strand priming using oligo-dT followed by random priming of the second strand is that amplified RNA will likely be biased toward the 3' end of the message since initial priming of the RNA is anchored with oligo-dT and thus potentially under-represents coding sequences found at the 5' of any given operon. However no bias was observed in the representation of microarray probes located within 500 bp of the 3' or 5' end of genes in the comparison of amplified to unamplified RNA. The variation between replicate amplifications using both methods was low as measured by yield, size distribution of product and gene expression pattern (correlation coefficients of 0.96–0.97 for oligo-dT replicate samples and 0.85–0.86 for replicate ADP amplifications); confirming that the products generated from these amplification systems were reproducible and suitable for microarray analyses.

The representative nature of the amplified products compared to unamplified RNA was measured by Spearman's rank correlation, and by applying fold change and signal/background thresholds. Correlation coefficients ranging from 0.79 to 0.84 were measured between amplified products from both amplification systems and unamplified RNA. This compares to other methods of amplification with correlation coefficients of amplified to unamplified of 0.44–0.75 for inputs of 10–5000 ng bacterial RNA [15]; or 0.73–0.99 for inputs of 10–100 ng [14]. Despite these relatively high correlation coefficients however, the number of genes over or under-represented in amplified compared to unamplified profiles for the same source RNA gives a better indication of method biasing. In this study the ADP method gave the least number of genes deviating from the expected ratio at the 500 ng input range (16%) but was otherwise similar at the 50 ng range (25%) to the oligo-dT method giving a 20–24% bias. Indeed other studies have applied statistical testing to identify between 0.6–2.6% [15] or 1.7–19% [9] of genes to have significant differential abundance between amplified and unamplified populations. We conclude therefore that there are sufficient differences in gene expression pattern to suggest that the direct comparison of amplified to unamplified RNA would be unwise.

The potential loss of expression data as a consequence of poor or inefficient gene specific amplification was investigated by comparing the number of genes flagged as absent in the microarray analyses. The number of undetected genes was higher after amplification using the ADP system compared to unamplified RNA, furthermore the number of predicted ADP primer-binding events did not appear to be a significant factor in the loss of detectable genes from the ADP amplified analysis. However lower signal intensities from a subset of genes using the ADP amplification method may lead to an increased number of aberrant expression ratios, and therefore decreased levels of significance when applied as part of down-stream data analysis. Correlation coefficients (0.90 – 0.98) between products amplified from different input amounts using the same amplification method demonstrated that the gene expression pattern of amplified RNA was similar, independent of input amount. This may be an important factor as variation in RNA input amount might be unavoidable in some experimental settings. The number of genes below the detection threshold however increased as RNA input into the amplification reactions decreased. This pattern of reduced sensitivity with decreasing input RNA was also observed using Whole Community RNA Amplification [9].

Little or no amplification products were detected when amplifying RNase treated samples, illustrating the minimal impact of contaminating genomic DNA on RNA amplification from the samples used in this study; however heavily contaminated samples may present difficulties and DNase treatment of RNA before amplification is essential. Additionally, product was amplified from 250 ng eukaryotic RNA using both amplification methods; in mixed RNA population amplifications however there was little or no effect of the contaminating eukaryotic RNA on bacterial transcriptional patterns measured using a *M. tuberculosis *microarray. This should allow for the successful transcriptome analysis of bacterial RNA extracted where the co-purification of at least equal quantities of contaminating eukaryotic RNA is unavoidable.

The amplification systems detailed in this study generate anti-sense aRNA, which may present difficulties if this product is to be labelled during cDNA synthesis of the amplified product for hybridisation to oligonucleotide microarrays (usually constructed using sense oligonucleotides). Rachman *et al*. [14] and Lawson *et al*. [15] have recently described amplification methods utilising a template switching primer to generate sense aRNA to overcome this issue. An alternative strategy would be the addition of biotin or amino allyl-linked nucleotides during amplification and then indirect incorporation of the fluorophores before hybridisation, or an additional RT step before direct labelling of product for microarray hybridisation. Both approaches have been successfully used in eukaryotic systems in our hands and elsewhere.

The bacterial amplification systems described here were tested on total *M. tuberculosis *RNA; the amplification of mycobacterial ribosomal RNA that may have overwhelmed the amplification reactions and subsequent hybridisations was not problematic. Whether this was due to the high tertiary structure of ribosomal RNA limiting primer binding, 3' modifications limiting polyA tailing or an excess of reagents is unknown. The major advantage of being able to successfully analyse gene expression patterns from amplified total RNA is the elimination of the requirement for mRNA purification from total RNA extractions.

## Conclusion

From this work we offer the following general recommendations for the amplification and microarray analysis of bacterial RNA: (A) remove contaminating DNA as it will likely be amplified alongside the bacterial RNA population; (B) remove contaminating eukaryotic RNA before amplification or assess the impact of co-purified eukaryotic RNA on amplified bacterial gene expression patterns; (C) amplify from similar input amounts of RNA to reduce variation between replicate samples; (D) amplify and hybridise samples from a single experiment together where possible; and crucially (E) do not compare amplified directly to unamplified RNA, or to RNA amplified using an alternative method.

The recognition of biologically relevant pathways in gene expression profiles derived from amplified RNA in the model system tested here confirms for the first time that the amplification of bacterial RNA may be used successfully in bacterial transcriptomics. RT-PCR validation will still be required for single gene inferences of biology, ideally from total RNA before amplification. However the advantages of microarray analyses, using changing patterns of transcription (through identification of co-regulated genes and the modified expression of defined functional categories) to interpret biologically significant scenarios, are still applicable using amplified RNA. This should facilitate the study of complex host-pathogen interactions that previously could not be investigated because of low bacterial numbers, an exhaustive number of timepoints/conditions, or low RNA extraction yields. In summary, we describe the RNA populations generated by *in vitro *transcript amplification following 2 methods of cDNA priming and define the limitations of this topical area of bacterial RNA amplification; the successful use of which will enable researchers to study previously intractable host-pathogen interactions where bacterial RNA is limiting.

## Methods

### Growth conditions and RNA extraction

*Mycobacterium tuberculosis *H37Rv was grown as agitated cultures (370 rpm) to mid-log phase at 37°C in Dubos liquid medium, supplemented with Dubos medium albumin. *M. tuberculosis *microaerophilic (NRP1) cultures were set up and cultured in a stirred model for 72 h according to Wayne and Hayes [17]. Mycobacterial RNA was extracted using the GTC/Trizol method as developed by Mangan *et al*. [20]; RNA was DNase-treated and purified using RNeasy columns (Qiagen). Total RNA was quantified using the NanoDrop ND-1000 Spectrophotometer (NanoDrop Technologies) and Agilent 2100 Bioanalyser (Agilent Technologies).

### Amplification-directed primer design

A minimal set of amplification-directed primers (ADP) were designed in an iterative fashion using an algorithm similar to that described by Talaat *et al*. [13] to bind within the first 500 bp (of the 3') of all genes annotated on the *M. tuberculosis *H37Rv [10], CDC1551 [11], and *M. bovis *AF2122/97 [12] genomes with a gene specific primer sequence of 7 nucleotides in length. Primer design entailed successive rounds of primer design followed by similarity searching, target sequence reduction and redesign to the remaining target sequences which resulted in the generation of 47 primers designed to bind to every annotated gene, with a mean of 7 primer hits/gene and 668 genes hit by each primer. A 5' anchor, a T7 polymerase binding sequence, and a random four nucleotide sequence (CGAAA-TTAATACGACTCACTATAGGGAGA-NNNN-7mer) were added to the primer sequence in order to integrate the genome-directed primers into an Eberwine-based amplification system. Further details of the ADP sequences can be found in Additional File 1. To assess the impact of the additional T7 nucleotide sequence on the specificity of the ADP primers, 5 μg *M. tuberculosis *H37Rv RNA was labelled for microarray hybridisation using reverse transcriptase with 2 μM ADP primer +/- the 5' anchor, T7 polymerase binding sequence, and random four nucleotide sequence. The products from five ADP (+/- T7 sequences) primed RT-labelling reactions were hybridised independently against genomic DNA to the *M. tuberculosis *microarray (as described below), and the signal from the RNA-derived products compared to measure the effect of the additional T7 sequence on ADP primer specificity.

### RNA amplification

50 μM *M. tb *amplification-directed primers (ADP) were used in the place of oligo-dT in the MessageAmp II eukaryotic amplification system (Ambion); the second priming strategy included an initial polyadenylation step (with *E. coli *polyA polymerase at 37°C for 15 minutes) followed by oligo-dT based amplification (MessageAmp II Bacteria, Ambion). Amplification reactions were conducted according to manufacturers instructions, with inputs of 500 ng, 50 ng and 5 ng total mycobacterial RNA. Single rounds of amplification were performed, with an IVT reaction of 16 hours at 37°C. Control reactions containing 500 ng *M. tb *H37Rv genomic DNA, 500 ng RNase-treated *M. tb *total RNA or water were also performed using both amplification systems. Amplifications were repeated in duplicate on two or more separate occasions. To determine the impact of contaminating eukaryotic RNA, 250 ng *M. tb *H37Rv total RNA +/- 250 ng eukaryotic total RNA, and 250 ng eukaryotic total RNA alone was amplified in triplicate using both amplification methods. The yield and size distribution of all amplified products was assessed spectrophotometrically at OD^260 ^and using the Agilent 2100 Bioanalyser (Agilent Technologies).

### Microarray analyses

A *M. tuberculosis *whole genome microarray consisting of 4410 gene specific PCR products (designed with minimal cross-homology) to the *M. tuberculosis *H37Rv [10], CDC1551 [11], and *M. bovis *AF2122/97 [12] genomes was utilised; this was generated by the Bacterial Microarray Group at St. George's [23], array accession number A-BUGS-23. Hybridisations were conducted as previously described [24] with, in the first and third studies (experimental design A), 5 μg Cy5-labelled cDNA derived from amplified or unamplified *M. tuberculosis *H37Rv RNA (aerated mid-log phase *in vitro *cultures) against 2 μg Cy3-labelled *M. tuberculosis *H37Rv genomic DNA (provided by Colorado State University). In the second comparison (experimental design B) 5 μg cDNA derived from amplified aerobic RNA was labelled with Cy3 and hybridised against 5 μg cDNA derived from amplified NRP1 (microaerophilic) RNA labelled with Cy5. The hybridised slides were scanned sequentially at 532 nm and 635 nm corresponding to Cy3 and Cy5 excitation maxima using the Affymetrix 428™ Array Scanner (MWG). Comparative spot intensities from the images were calculated using Imagene 5.5 (BioDiscovery), and imported into GeneSpring GX™ 7.2 (Agilent Technologies) for further analysis. The array data were normalised to the 50th percentile of all genes detected to be present on the array. In the first microarray experiment (A) the expression of all *M. tuberculosis *H37Rv genes were analysed (with no additional filtering). Spearman's rank correlations and over/under-represented genes were generated from combining replicate hybridisations of each condition (with a minimum of 2 and maximum of 6 replicates/condition). In the second (B) aerobic vs. NRP1 experiment two independent amplifications were conducted and hybridised in duplicate. These 4 hybridisations were combined together before significantly differentially regulated genes were identified between aerobic and microaerophilic (NRP1) conditions using a t-test p-value < 0.05 with Benjamini and Hochberg multiple testing correction and a > 2.5 fold cutoff. A minimum of 5 replicate hybridisations were conducted with unamplified aerobic and NRP1 RNA before defining statistically significant genes as above. The hypergeometric distribution was used to determine if previously published functional categories of genes were significantly enriched in the amplified comparisons [25]. Fully annotated raw and filtered microarray data has been deposited in BμG@Sbase (accession number: E-BUGS-42) [26] and also ArrayExpress (accession number: E-BUGS-42).

## Abbreviations

ADP, Amplification-Directed Primers; NRP1, Non-Replicating Persistence Stage 1; IVT, *in vitro *transcription; aRNA, Amplified RNA.

## Authors' contributions

SJW designed the study and carried out the amplifications and amplified RNA microarray analyses and also drafted the manuscript. KL designed the amplification-directed primer set, helped analyse the microarray datasets and draft the manuscript. CS extracted the RNA and performed the unamplified RNA microarray analysis. PDB conceived of the study and helped draft the manuscript. All authors read and approved the final manuscript.

## Supplementary Material

Additional file 1 47 amplification-directed primer sequences. The nucleotide sequences of the amplification-directed primers designed and utilised in this study. Click here for file
