# Supplementary material for: Microarray analysis of defined Mycobacterium tuberculosis populations using RNA amplification strategies
Source: BMC Genomics. 2008 Feb 25;9:94. doi: 10.1186/1471-2164-9-94 (PMC2276497; doi:10.1186/1471-2164-9-94)
Supplement: Additional file 1 — 47 amplification-directed primer sequences. The nucleotide sequences of the amplification-directed primers designed and utilised in this study. [file 1471-2164-9-94-S1.pdf]

Additional File 1 - Waddell *et al* ., 2008
47 amplification-directed primer sequences

ADP Primer	Primer Sequence (5'-3')
T7N4_TB1	CGAAATTAATACGACTCACTATAGGGAGANNNNCGGCGGC
T7N4_TB3	CGAAATTAATACGACTCACTATAGGGAGANNNNGGCCAGC
T7N4_TB4	CGAAATTAATACGACTCACTATAGGGAGANNNNGTCGGCG
T7N4_TB5	CGAAATTAATACGACTCACTATAGGGAGANNNNCGCCGGC
T7N4_TB6	CGAAATTAATACGACTCACTATAGGGAGANNNNGTCGTCG
T7N4_TB7	CGAAATTAATACGACTCACTATAGGGAGANNNNCGCCAGC
T7N4_TB8	CGAAATTAATACGACTCACTATAGGGAGANNNNGCCGTCG
T7N4_TB9	CGAAATTAATACGACTCACTATAGGGAGANNNNCACCGCG
T7N4_TB10	CGAAATTAATACGACTCACTATAGGGAGANNNNCAGCAGC
T7N4_TB11	CGAAATTAATACGACTCACTATAGGGAGANNNNCGGTGCC
T7N4_TB12	CGAAATTAATACGACTCACTATAGGGAGANNNNGCCACCG
T7N4_TB13	CGAAATTAATACGACTCACTATAGGGAGANNNNCGCGCCG
T7N4_TB14	CGAAATTAATACGACTCACTATAGGGAGANNNNGCGGCCG
T7N4_TB15	CGAAATTAATACGACTCACTATAGGGAGANNNNGGCGTCG
T7N4_TB16	CGAAATTAATACGACTCACTATAGGGAGANNNNCAGCGCC
T7N4_TB18	CGAAATTAATACGACTCACTATAGGGAGANNNNGATGGTG
T7N4_TB19	CGAAATTAATACGACTCACTATAGGGAGANNNNTCGCCGC
T7N4_TB20	CGAAATTAATACGACTCACTATAGGGAGANNNNACGCGGC
T7N4_TB22	CGAAATTAATACGACTCACTATAGGGAGANNNNCAGCTCG
T7N4_TB23	CGAAATTAATACGACTCACTATAGGGAGANNNNGGCGCCG
T7N4_TB24	CGAAATTAATACGACTCACTATAGGGAGANNNNCAGCCGC
T7N4_TB25	CGAAATTAATACGACTCACTATAGGGAGANNNNGCCGCCC
T7N4_TB26	CGAAATTAATACGACTCACTATAGGGAGANNNNGATCGGC
T7N4_TB27	CGAAATTAATACGACTCACTATAGGGAGANNNNGTCCTCG
T7N4_TB28	CGAAATTAATACGACTCACTATAGGGAGANNNNGCTCGCG
TBV2_3	CGAAATTAATACGACTCACTATAGGGAGANNNNCGCCGAG
TBV2_5	CGAAATTAATACGACTCACTATAGGGAGANNNNGGTGCGG
TBV2_6	CGAAATTAATACGACTCACTATAGGGAGANNNNGACGACG
TBV2_7	CGAAATTAATACGACTCACTATAGGGAGANNNNGCTCGAG
TBV2_8	CGAAATTAATACGACTCACTATAGGGAGANNNNCGACGTG
TBV2_9	CGAAATTAATACGACTCACTATAGGGAGANNNNCGCCAAG
TBV2_10	CGAAATTAATACGACTCACTATAGGGAGANNNNCAGCGTC
TBV2_11	CGAAATTAATACGACTCACTATAGGGAGANNNNACGACCC
TBV2_12	CGAAATTAATACGACTCACTATAGGGAGANNNNTCGCGGT
TBV2_13	CGAAATTAATACGACTCACTATAGGGAGANNNNCACCAGC
TBV2_14	CGAAATTAATACGACTCACTATAGGGAGANNNNCGCCCAC
TBV2_15	CGAAATTAATACGACTCACTATAGGGAGANNNNTCACCGA
TBV2_16	CGAAATTAATACGACTCACTATAGGGAGANNNNCATCGGC
TBV2_17	CGAAATTAATACGACTCACTATAGGGAGANNNNCGAGCCG
TBV2_18	CGAAATTAATACGACTCACTATAGGGAGANNNNCGGGCGC
TBV2_19	CGAAATTAATACGACTCACTATAGGGAGANNNNAGCCGGC
TBV2_20	CGAAATTAATACGACTCACTATAGGGAGANNNNCACAACG
TBV2_21	CGAAATTAATACGACTCACTATAGGGAGANNNNACCCGGC
TBV3_2	CGAAATTAATACGACTCACTATAGGGAGANNNNCCGGTGG
TBV3_3	CGAAATTAATACGACTCACTATAGGGAGANNNNCCGACTC
TBV4_1	CGAAATTAATACGACTCACTATAGGGAGANNNNACGTCGG
TBV4_2	CGAAATTAATACGACTCACTATAGGGAGANNNNAACCGGT
